# Kawasaki disease with cardiac involvement revealed by acute pancreatitis in an adult: a rare case report

**DOI:** 10.1186/s43044-025-00649-9

**Published:** 2025-05-26

**Authors:** Leila BARAKAT, Meryem HABOUB, Safaa MHABER, Khadija ECHCHILALI, Mina MOUDATIR, Salim AROUS, Mohamed Ghali BENOUNA, Rachida HABBAL, Abdenasser DRIGHIL, Hassan EL KABLI

**Affiliations:** 1 University of Hassan II Casablanca, Casablanca, Morocco; 2 Laboratory of Clinical Immunology, Allergy and Inflammation (LICIA), Laboratory of Clinical Immunology, Allergy and Inflammation (LICIA), Morocco; 3 Ibn Rochd University Hospital Center, Ibn Rochd University Hospital Center, Morocco

**Keywords:** Kawasaki disease, Acute pancreatitis, Coronary aneurysm, Systemic vasculitis, Intravenous immunoglobulins

## Abstract

**Background:**

Kawasaki disease (KD) is a rare systemic inflammatory disease primarily affecting children under 5 years of age, with very few cases reported in adults. This condition is characterized by potential coronary involvement, including aneurysms and gastrointestinal manifestations, such as pancreatitis.

**Case presentation:**

A 21-year-old man presented with a febrile rash, fever, conjunctivitis, and acute pancreatitis. Imaging revealed coronary artery aneurysms. Treatment with intravenous immunoglobulins, aspirin, and corticosteroids led to significant clinical improvement.

**Conclusions:**

This case underscores the rare occurrence of Kawasaki disease in adults, particularly with both pancreatic and coronary artery involvement. The effective use of immunoglobulins and corticosteroids highlights the importance of early diagnosis and treatment in managing this rare condition in adults.

**Supplementary Information:**

The online version contains supplementary material available at 10.1186/s43044-025-00649-9.

## Background

Kawasaki disease (KD) is a rare systemic inflammatory disease that primarily affects children under 5 years of age [1]. The incidence of adult-onset KD is extremely low, with only approximately 100 cases reported to date [2]. Coronary involvement is the most common complication of the disease and manifests as aneurysm, calcification, or vascular stenosis [3]. Gastrointestinal manifestations are uncommon in Kawasaki disease, and pancreatitis has been exceptionally reported in adults [4].

### Case presentation

**Fig. 1:**
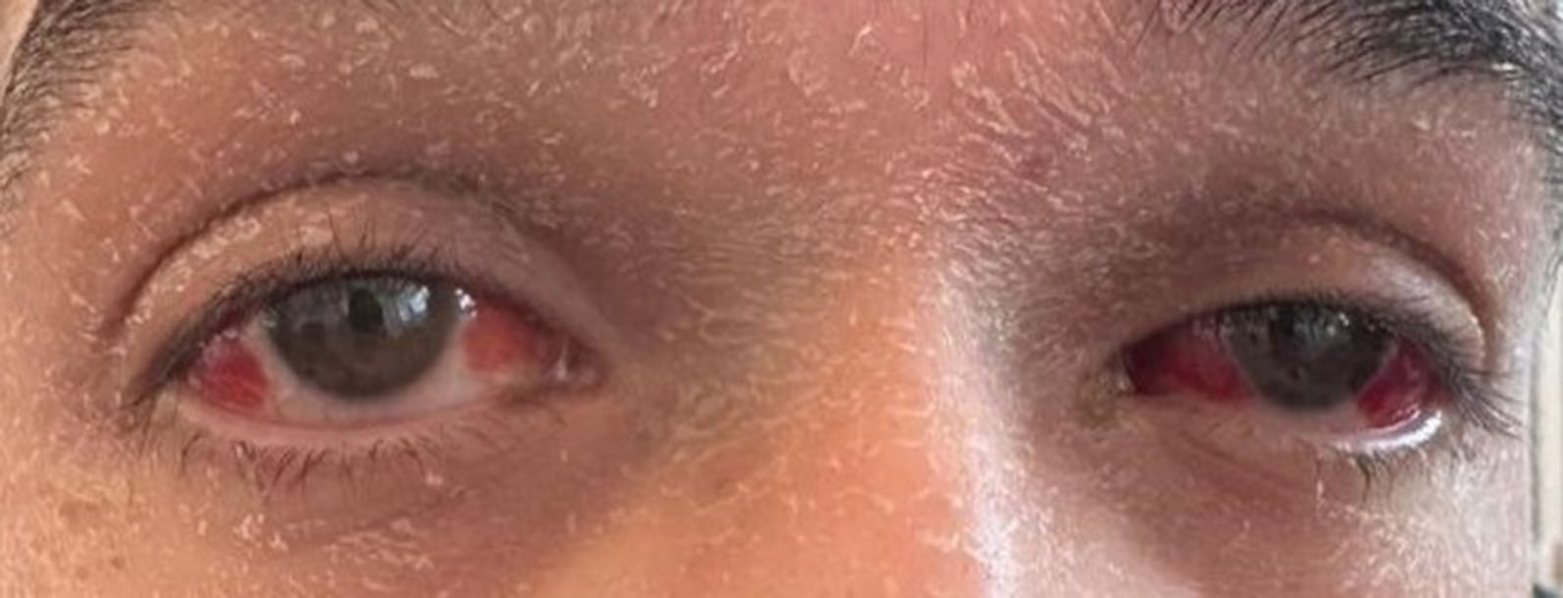
Bilateral conjunctivitis

**Fig. 2:**
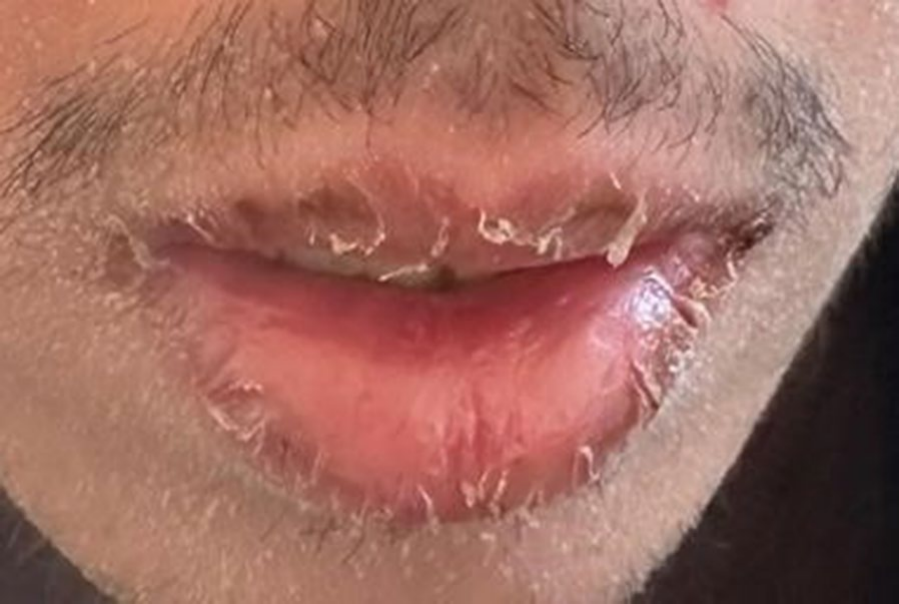
Cheilitis

**Fig. 3:**
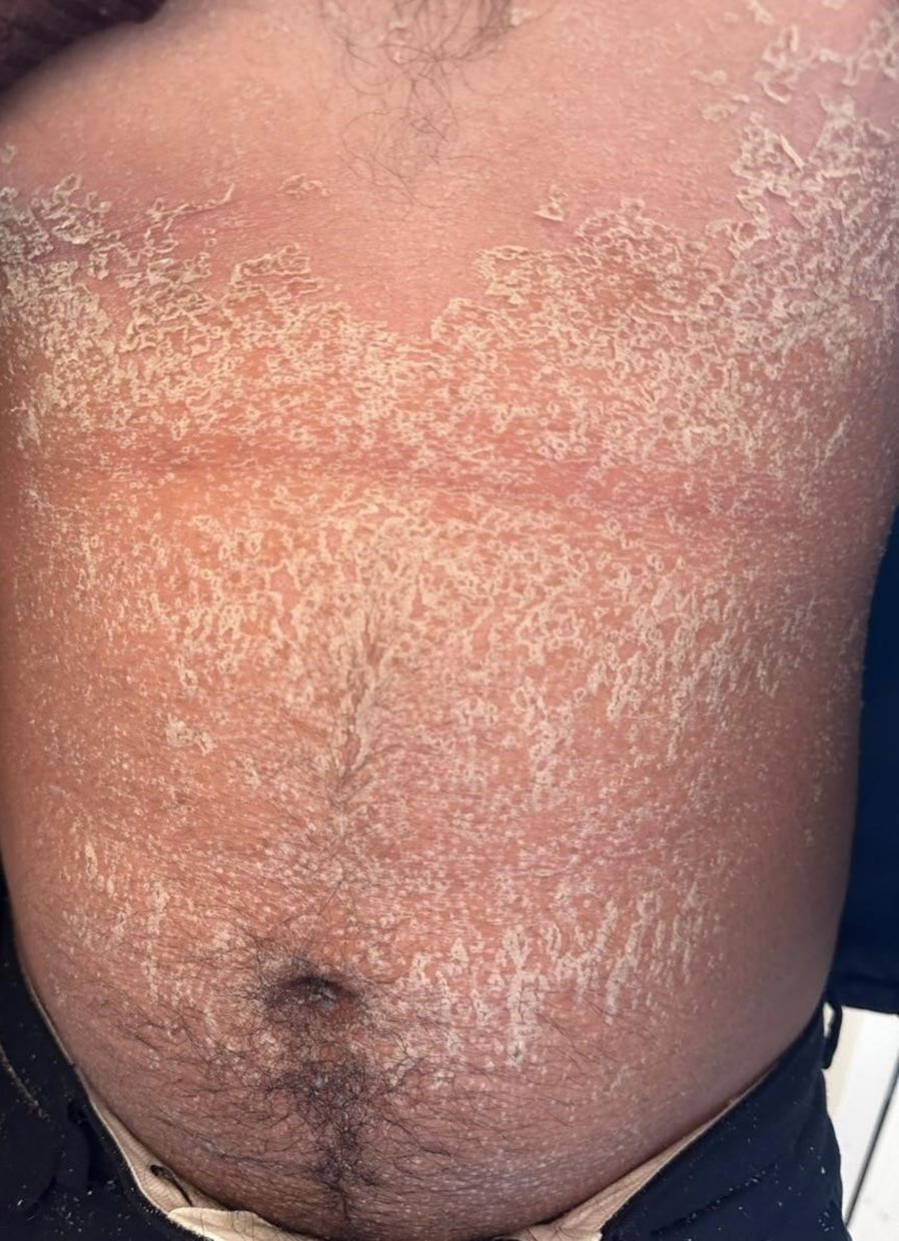
Diffuse desquamation of the trunk

**Fig. 4:**
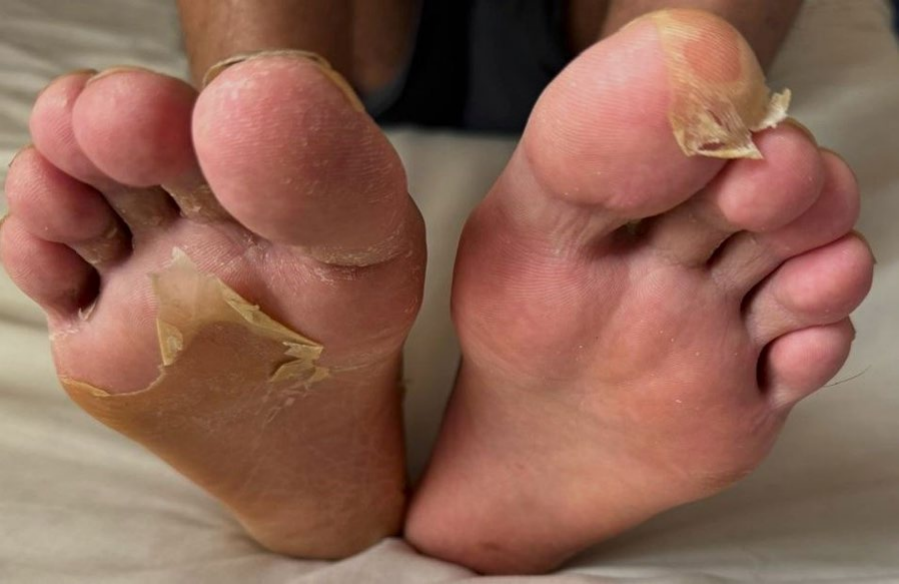
Desquamation of extremities

**Fig. 5:**
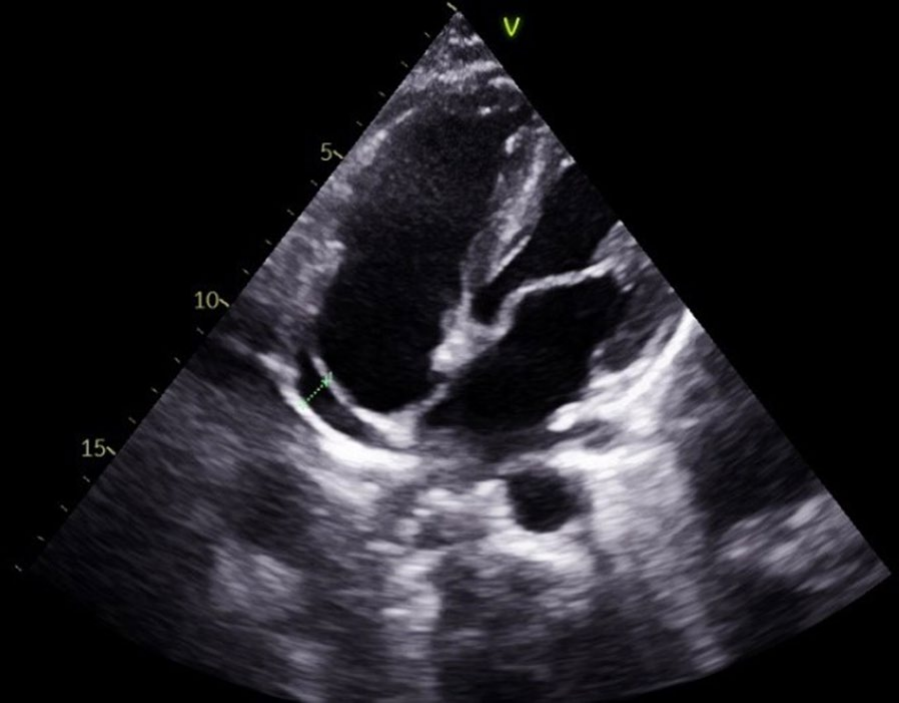
Mild pericardial effusion behind the right atrium

**Fig. 6:**
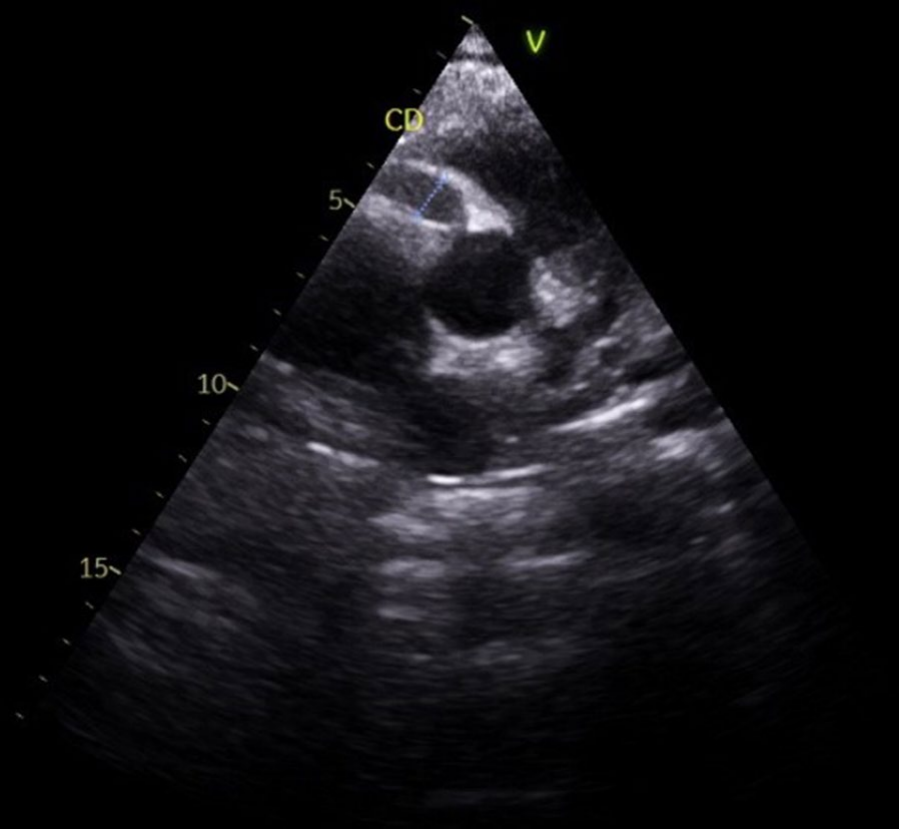
Dilation of the proximal right coronary artery measuring 10 mm

**Fig. 7:**
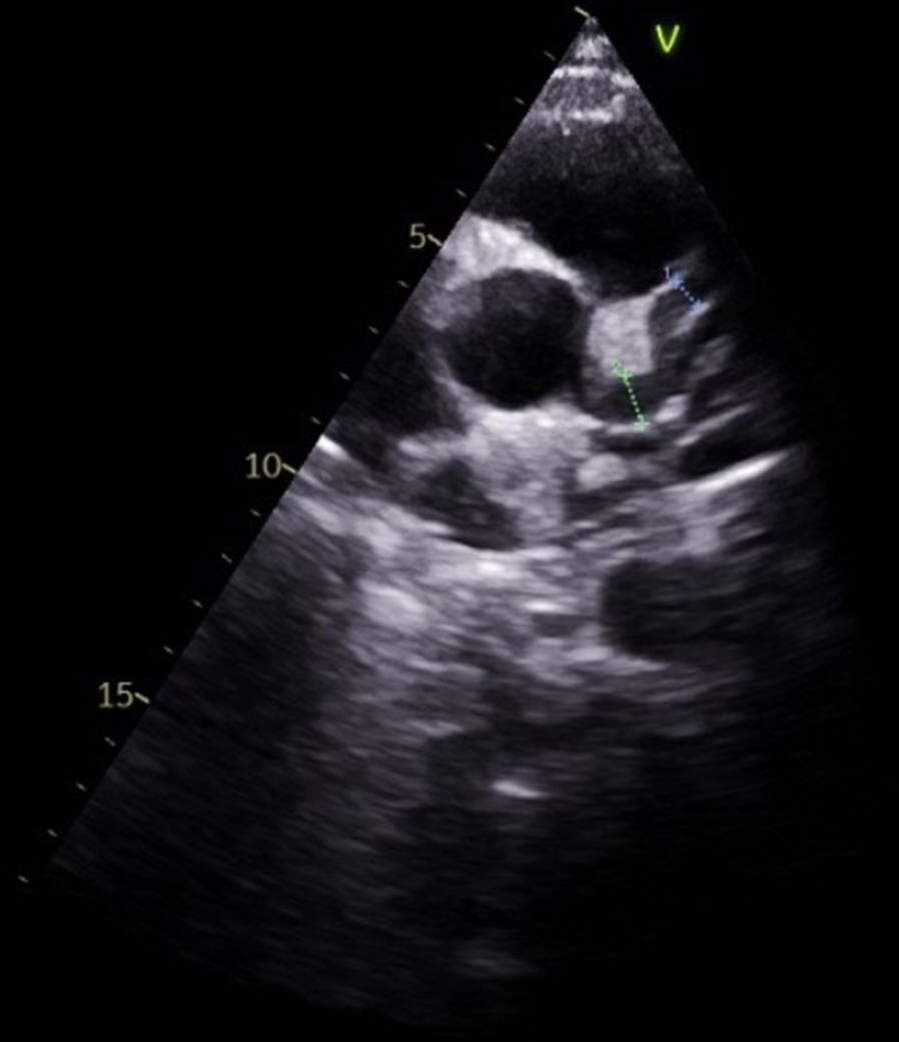
Dilation of the left anterior descending artery measuring 6 mm

**Fig. 8:**
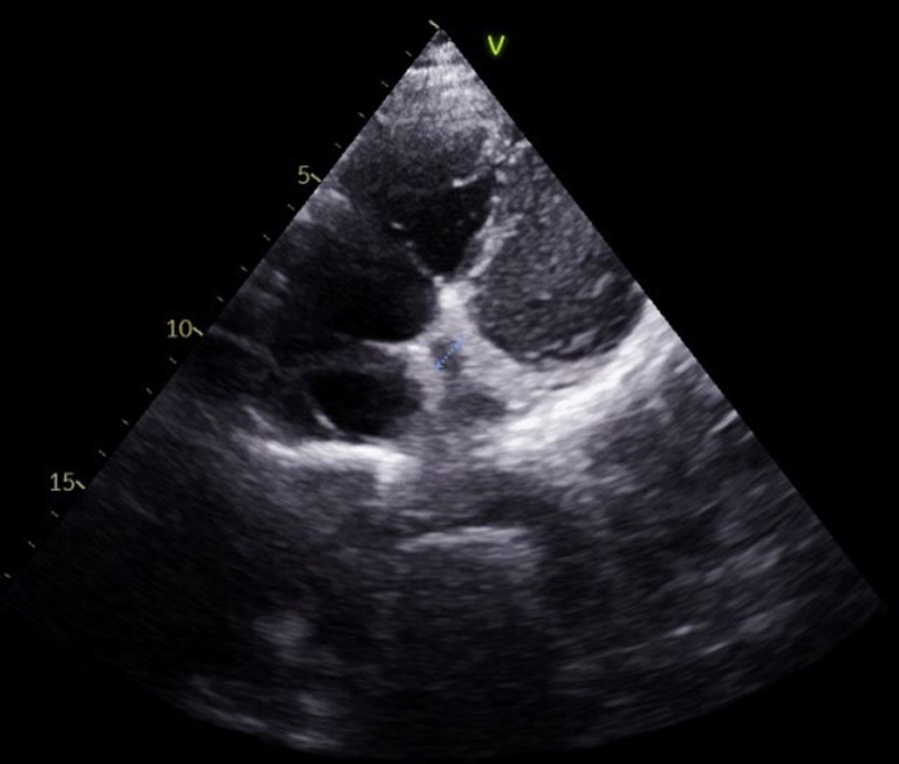
Dilation of the circumflex artery measuring 8 mm

**Fig. 9:**
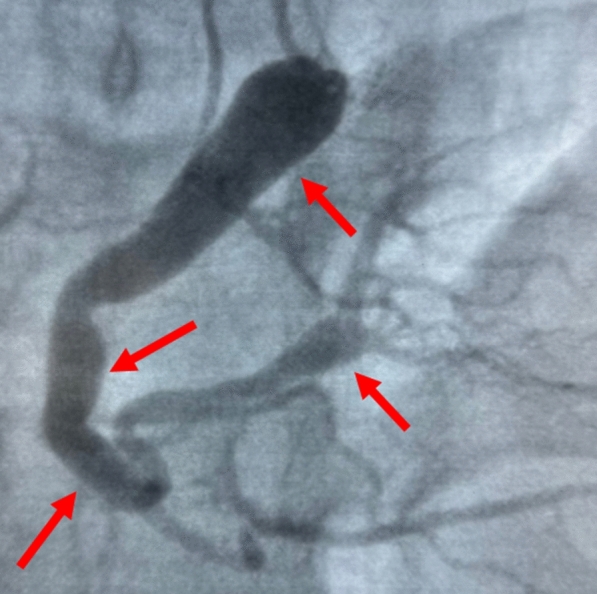
Images in the strict cranial view revealed the right coronary artery with multiple aneurysms in all segments (red arrows)

- A 21-year-old man with no significant medical history, recent medication intake, or infectious episode was referred to the internal medicine department for an etiological assessment of a febrile rash. The clinical presentation had been evolving for 20 days and was initially characterized by a febrile maculopapular rash at 39 °C, with the fever persisting throughout the entire 20-day period and showing only partial response to antipyretic, rapidly accompanied by edema of the extremities, bilateral conjunctivitis (Fig. 1), and cheilitis (Fig. 2). The clinical presentation was further complicated by the onset of acute pancreatitis 6 days later, manifested by abdominal pain, a lipase level elevated to 6 times the normal value, and a computed tomography (CT) scan showing features consistent with stage C pancreatitis. An abdominal CT angiography revealed no aneurysmal dilatation of the mesenteric vessels. Laboratory tests revealed elevated inflammatory markers with a C-reactive protein (CRP) level of 160 mg/L (normal < 5 mg/L). Complete blood count revealed no anemia, but showed leukocytosis at 12.000/mm^3^ (normal 4.000–10.000 /mm^3^) predominantly neutrophilic, along with thrombocytosis reaching 520.000/mm^3^ (normal 150.000–400.000 /mm^3^). The infectious workup was negative, including testing for severe acute respiratory syndrome coronavirus 2 (SARS-CoV-2), parvovirus B19, Epstein–Barr virus (EBV), and human immunodeficiency virus (HIV). The evolution was marked by diffuse desquamation of the trunk (Fig. 3) and extremities (Fig. 4).
- Transthoracic echocardiography showed normal ventricles and atria, no valvular heart disease, no aortic dilatation, mild pericardial effusion behind the right atrium (Fig. 5), and a coronary artery aneurysm. The proximal right coronary artery measured 10 mm (normal 3-4 mm) (Fig. 6), the left main coronary artery was dilated to 9 mm (normal 4-5 mm), the left anterior descending (LAD) was dilated to 6 mm (normal 3-4 mm) (Fig. 7), and the circumflex artery (CX) was dilated to 8 mm (normal 2.5-4 mm) (Fig. 8). Coronary angiography revealed images in the left anterior oblique (LAO) cranial view at 30°/30° showing an aneurysmal common trunk and proximal left anterior descending artery. Images in the right anterior oblique (RAO) caudal view at 30°/30° showed a slightly aneurysmal circumflex artery. Images in the strict cranial view revealed the right coronary artery with multiple aneurysms in all segments (red arrows in Fig. 9).
- Kawasaki disease (KD) was diagnosed, and treatment was initiated with intravenous methylprednisolone at a dose of 1 g per day for three consecutive days. On the fourth day, the patient received intravenous immunoglobulins (IVIG) in combination with aspirin. The clinical course was favorable, with resolution of fever (apyrexia) occurring 48 h after the initiation of corticosteroid therapy. The inflammatory syndrome progressively resolved, with CRP levels decreasing to below 5 mg/L and the lipase level was normal after 15 days of treatment. The complete blood count also returned to normal, with normalization of white blood cell count and platelet count. At one-year follow-up, the patient remained clinically stable. Transthoracic echocardiography performed one year after the acute episode showed persistent coronary aneurysms with no significant change in size or morphology.

## Discussion

- Kawasaki disease (KD) is a systemic inflammatory condition of uncertain origin that predominantly affects children under five years of age. The exact cause of Kawasaki disease remains unknown, although seasonal episodes, particularly in summer and winter, suggest the possibility of an infectious trigger. Diagnostic criteria for adult Kawasaki disease have also been proposed [5].
- The diagnosis is probable if an individual presents with unexplained persistent fever for more than 5 days with an onset age of first manifestations over 18 years, along with coronary involvement and 3 out of 5 criteria, which are as follows: i) exanthema; ii) oral mucosal changes; iii) extremity changes; iv) conjunctivitis; and v) lymphadenopathy. Our patient met four criteria in addition to prolonged fever and coronary involvement, which was sufficient to establish a diagnosis of Kawasaki disease.
- Pericarditis is a common but underreported manifestation. It is typically mild and asymptomatic [6]. Digestive involvement was also possible. Common gastrointestinal signs include abdominal pain, diarrhea, hepatitis, vomiting, and gallbladder hydrops; jaundice and pancreatitis are less common [7]. Histological pancreatic involvement has been commonly observed in autopsy reports, but clinical pancreatitis remains rare [8]. Pancreatitis in Kawasaki disease is characterized by vasculitis of the medium-sized arteries and veins. The beneficial effects of IV Ig in Kawasaki disease complicated by pancreatitis have been previously explained by the resolution of vasculitis in pancreatic blood vessels [9]. A literature review highlighted that pancreatitis can precede the onset of Kawasaki disease symptoms, be part of the clinical picture of the disease, or even occur several days after IgIV administration. Abdominal pain is the most common symptom of pancreatic involvement, although some patients remain asymptomatic and only exhibit elevated lipase levels [10]. Only two cases of Kawasaki disease in adults with pancreatitis have been reported in the literature [8, 11], and to date, no cases have been described in adults presenting with both pancreatitis and coronary artery aneurysm.
- The recommended treatment regimen for Kawasaki disease is a single infusion of 2 g/kg of IVIG, which may be repeated in cases of resistance defined by persistent or recurrent fever after 36 h [3]. Treatment should be initiated promptly, preferably before the 10th day following the onset of the disease, as long as the inflammation persists. However, diagnosis within the first 10 days of the disease is rare and often results in delayed treatment initiation [12]. Corticosteroids are commonly prescribed for most vasculitides because of their rapid action, potent anti-inflammatory properties, and generally positive outcomes. Their use in Kawasaki disease treatment is more debated, but emerging data suggest that patients at high risk of developing coronary artery aneurysms may benefit from early initiation of corticosteroids in addition to IVIG and aspirin [1]. Although the diagnosis was made 20 days after the onset of the first symptoms, a bolus of methylprednisolone was administered to our patient because of the presence of two severe manifestations, pancreatic and cardiac, in combination with immunoglobulins and aspirin.

## Conclusion

This case highlights the exceptional occurrence of Kawasaki disease in an adult, marked by simultaneous pancreatic and coronary artery involvement. The successful administration of intravenous immunoglobulins and corticosteroids resulted in significant clinical improvement, emphasizing the need for increased awareness and prompt recognition of this rare condition in adult patients.

## Competing interest

The authors declare no competing interests.

## Supplementary Information

Supplementary file1.

## Data Availability

No datasets were generated or analyzed during the current study.

## Abbreviations

KD: Kawasaki disease
CT: Computed tomography
CRP: C-reactive protein
CX: Circumflex artery
LAO: Left anterior oblique
LAD: Left anterior descending
RAO: Right anterior oblique
IVIG: Intravenous immunoglobulins
